# Heatwave winners and losers: cryptic coral holobionts differ in thermal tolerance

**DOI:** 10.1098/rspb.2026.0890

**Published:** 2026-06-03

**Authors:** Zoe Meziere, Ilha Byrne, Andrew Khalil, Iva Popovic, Adriana Humanes, James R. Guest, Cheong Xin Chan, Cynthia Riginos, Katrina McGuigan

**Affiliations:** ^1^ School of the Environment, University of Queensland, St Lucia, Queensland, Australia; ^2^ Australian Institute of Marine Science, Townsville, Queensland, Australia; ^3^ School of Natural and Environmental Sciences, Newcastle University, Newcastle upon Tyne, UK; ^4^ Australian Centre for Ecogenomics, University of Queensland, St Lucia, Queensland, Australia

**Keywords:** coral bleaching, common garden, adaptation, marine heatwave, cryptic species

## Abstract

Extreme climatic events are reshaping ecosystems worldwide as individual organisms vary markedly in their ability to withstand these disturbances. Deciphering patterns of persistence on local scales is therefore critical for predicting biodiversity trajectories under intensifying climate extremes. In this study, we examined variation in thermal stress responses among individuals of the coral *Stylophora pistillata* species complex during a heatwave at Heron Island Reef, Australia. More than half of the focal coral colonies died on the reef, and survival of coral fragments maintained under *ex situ* common thermal stress conditions was significantly correlated with the survival of their source colony. This demonstrates that survival differences result largely from biological factors rather than differential thermal exposure across reef habitats. Under common garden conditions, we observed striking differences in bleaching severity and survival times among three sympatric cryptic taxa and their highly host-specific symbiont community. Within the most locally common taxon, corals from historically warmer and more seasonally variable reef habitats seem more susceptible to bleaching, contrary to expectations. Together, these results reveal how biological differences among cryptic taxa and among individuals can shape coral responses during a heatwave and advance our understanding of coral vulnerability in a rapidly warming world.

## 1. Introduction

Why do some individuals survive extreme climatic events while others do not? Organisms at a given location are rarely affected equally by acute environmental perturbations such as droughts, heatwaves or floods. Understanding the factors driving this variation has never been more urgent given the increasing frequency, intensity and duration of climate anomalies [1,2]. To assess vulnerability to an extreme event, we need to differentiate between the factors that determine sensitivity and the factors that govern exposure [3]. In this framework, sensitivity is driven by traits that are intrinsic to a species, whereas exposure reflects extrinsic factors determined by local habitat effects [4].

Species inhabiting the same ecosystem often have different sensitivity to a given environmental stress, resulting in different survival outcomes following an extreme event. Large-bodied, short-lived, low-dispersal and highly specialized species, for example, have been theorized to be more susceptible [5]. While tolerant species will tend to survive, more sensitive species might exhibit higher risks of local population decline or extinction [6]. As a result, climate extremes are rapidly altering species richness and community compositions across many ecosystems [2]. At the same time, most ecosystems are structurally complex with different (micro)habitats, and this fine-scale environmental variation can buffer organisms from extreme conditions (reviewed in [7]). For instance, leaf litter and tree holes can regulate temperature and moisture regimes within a rainforest and have been shown to become local thermal refugia during hot periods [8]. As a result, individuals inhabiting adjacent habitats might experience different exposure to a given climatic event and consequently exhibit different phenotypic responses. Therefore, survival under climate extremes can depend on both biological and environmental factors. The rarity and unpredictability of extreme climate events present a major challenge to disentangling these factors [9], yet this information is important for predicting and managing biodiversity under intensifying climate change.

Tropical coral reefs provide a striking example of ecosystems increasingly threatened by extreme climatic events, particularly marine heatwaves [10,11]. Marine heatwaves can last several weeks and significant variation in thermal stress responses are often observed among coral colonies inhabiting the same reef (e.g. [10,12]). Intrinsically, not all coral species exhibit the same sensitivity to thermal stress. For example, a coral species’ morphotype (e.g. [13,14]), tissue thickness (e.g. [13]), or ability to feed heterotrophically (e.g. [15,16]) may impact its thermal sensitivity. Although morphologically similar species are often assumed to be ecologically equivalent [17], growing evidence indicates that morphologically cryptic coral species can also exhibit different bleaching responses (e.g. [18,19]).

Additionally, the coral-algal symbiosis is an important intrinsic factor shaping holobiont fitness. Coral growth and calcification largely relies on the translocation of compounds from photosynthetic dinoflagellates of the family *Symbiodiniaceae* [20]. Coral species differ in how this partnership is established: while some acquire symbionts from the environment, others transmit them maternally, a mechanism that is expected to result in greater symbiont-host specificity [21]. Similarly, host coral species can form strict associations with only one *Symbiodiniaceae* taxon or associate with multiple taxa. This variation in symbiont acquisition matters because some symbiont taxa confer greater heat tolerance to the holobiont than others (e.g. [22,23]), and a more flexible symbiosis may allow for the acquisition of more heat-tolerant symbionts during heat stress [24]. When symbiosis is disrupted, the resulting loss of photosynthetic symbionts leads to coral bleaching and ultimately death if symbiosis is not restored [25]. However, symbiont community composition within a coral colony cannot be assessed through visual surveys and the coral microbiome therefore represents another hidden player that could explain differential coral survival during thermal stress.

Importantly, heatwaves do not uniformly affect all parts of the reef and thermal stress exposure therefore varies across habitats. For example, reef flats and lagoons are subject to strong tidal oscillations and can reach very high temperatures on low tides. While locations exposed to currents and wave action typically remain cooler, neighbouring locations sheltered from constant water movement are warmer [26,27]. As a result, some reef habitats might experience disproportionate levels of heat exposure during a heatwave, which could also explain why corals inhabiting these habitats are more affected. Notably, variation in thermal exposure across habitats can confound observations of species-specific responses during a heatwave if the species of interest occupy different habitats. In particular, this might be a challenge when studying thermal sensitivity differences in coral species complexes, as cryptic species often inhabit distinct environments (reviewed in [28]).

In addition to intrinsic differences in thermal sensitivity among species, conspecific individuals occupying environmentally distinct habitats might also differ in environmental sensitivity if they are locally acclimatized or adapted. While long-term acclimatization can induce persistent physiological adjustments in response to the local environmental conditions, natural selection acts over multiple generations to favour genotypes with optimal phenotypes [29,30]. Both processes result in phenotypes (either plastic or genetic) that match their environment and could therefore explain variation in intra-specific responses among habitats during an extreme climatic event [31,32]. Previous studies have found evidence for fine-scale thermal sensitivity differences among conspecific coral colonies under experimental heat stress (e.g. [33,34]). However, fewer studies have related this thermal sensitivity variation to habitat-specific historical thermal regimes, so it remains unclear whether it is adaptive (but see [35–37]).

Advancing our understanding of variation in coral thermal stress responses requires separating intrinsic factors affecting sensitivity (i.e. identity and genotype of the host and the symbionts) from the extrinsic factor of thermal stress exposure. Common garden experiments have long been used to test intrinsic effects, by growing or keeping individuals originating from different habitats in the same environment [38,39]. To further clarify the effect of environmental history on the phenotypes expressed under common conditions, we can determine how much of the phenotypic variation can be attributed to environmental variation among the natal habitats [40,41]. Although coral research has extensively utilized experimental designs where coral fragments are all exposed to the same thermal stress, these experiments have not typically applied heat stress regimes that reflect realistic heatwave conditions [42], have not considered variation in recovery trajectories following the heat stress, and it remains unclear whether *ex situ* responses of fragments reflect whole-colony responses on the reef [43].

In this study, we took advantage of a real-time marine heatwave to investigate thermal sensitivity variation among *Stylophora pistillata* corals, integrating *in situ* observations with *ex situ* common garden experiment. *S. pistillata* is a brooding coral with metre-scale larval dispersal [44], an early coloniser following disturbances [45], and was recently identified as a complex of cryptic and sympatric species across the Great Barrier Reef [46]. *S. pistillata* is categorized among the most thermally susceptible corals (e.g. [13,47]), however, previous studies did not account for the existence of cryptic taxa. Other studies have also documented high intra-specific bleaching variation among *S. pistillata* colonies in the field (e.g. [48]), consistent with cryptic species that differ in thermal stress sensitivity. These attributes make *S. pistillata* an ecologically interesting and tractable system to investigate response variation within and among species under thermal stress. Our goals were to (i) quantify thermal stress response variation among coral colonies during a marine heatwave, (ii) test the importance of intrinsic sensitivity differences between cryptic coral holobionts, and (iii) compare coral colony survival under common garden and *in situ* to assess the effect of habitat-specific thermal stress exposure. We predicted both among-species and within-species thermal stress response variation, reflecting differences in intrinsic sensitivity and local adaptation to contrasting thermal habitats.

## 2. Methods

### (a) Bleaching event, coral collection and common garden experiment

In 2024, a mass coral bleaching affected the Southern Great Barrier Reef (GBR) of Australia [49]. At Heron Island Reef, the heatwave lasted from mid-January to May and resulted in coral bleaching across 65% of the reef slope [50]. Shortly after the onset of the heatwave, 3–7 February inclusive, we sampled 80 *S*. *pistillata* colonies from 10 locations (4–12 colonies per location) across Heron Island Reef (electronic supplementary material, tables S1 and S2) following depth transects from 12 to 2 m (Marine Parks Permit number G21/44774.1). Each coral was photographed and tagged, and we collected three 5 cm length fragments (used in the common gardens; *n* = 240) using a hammer and a chisel and one 2 cm^2^ tissue sample (used for DNA sequencing; *n* = 80) using pliers. Within 2 h after collection, the fragments were glued onto labelled aragonite substrata and placed in aquaria (electronic supplementary material, figure S1). Tissue samples were fixed in absolute ethanol and then placed in a −20°C freezer. Sampled colonies were revisited 2.5 months after initial collections (15–19 April inclusive), when temperatures had returned below historical maximum monthly mean, to assess their survival.

Common garden exposure lasted 84 days (3 February–26 April), largely spanning the heatwave duration. Seawater was sourced at 15 m depth between Heron Island Reef and Wistari Reef and was pre-filtered to exclude particles larger than 3 mm. We created a semi flow-through system with two outdoor tables, three sumps per table and two 70 l aquarium tanks per sump (electronic supplementary material, figure S1). Water temperature was not manipulated and followed natural diel fluctuations (figure 1D) with similar temperature profiles among tanks (mean s.d. = 0.1°C; electronic supplementary material, figure S2). On coral reefs, the intensity and duration of heat stress is commonly quantified in degree heating week (DHW; °C-wk). We calculated DHW prior to the start of the common garden experiment and for the duration of the experiment (see electronic supplementary material).

**Figure 1.**
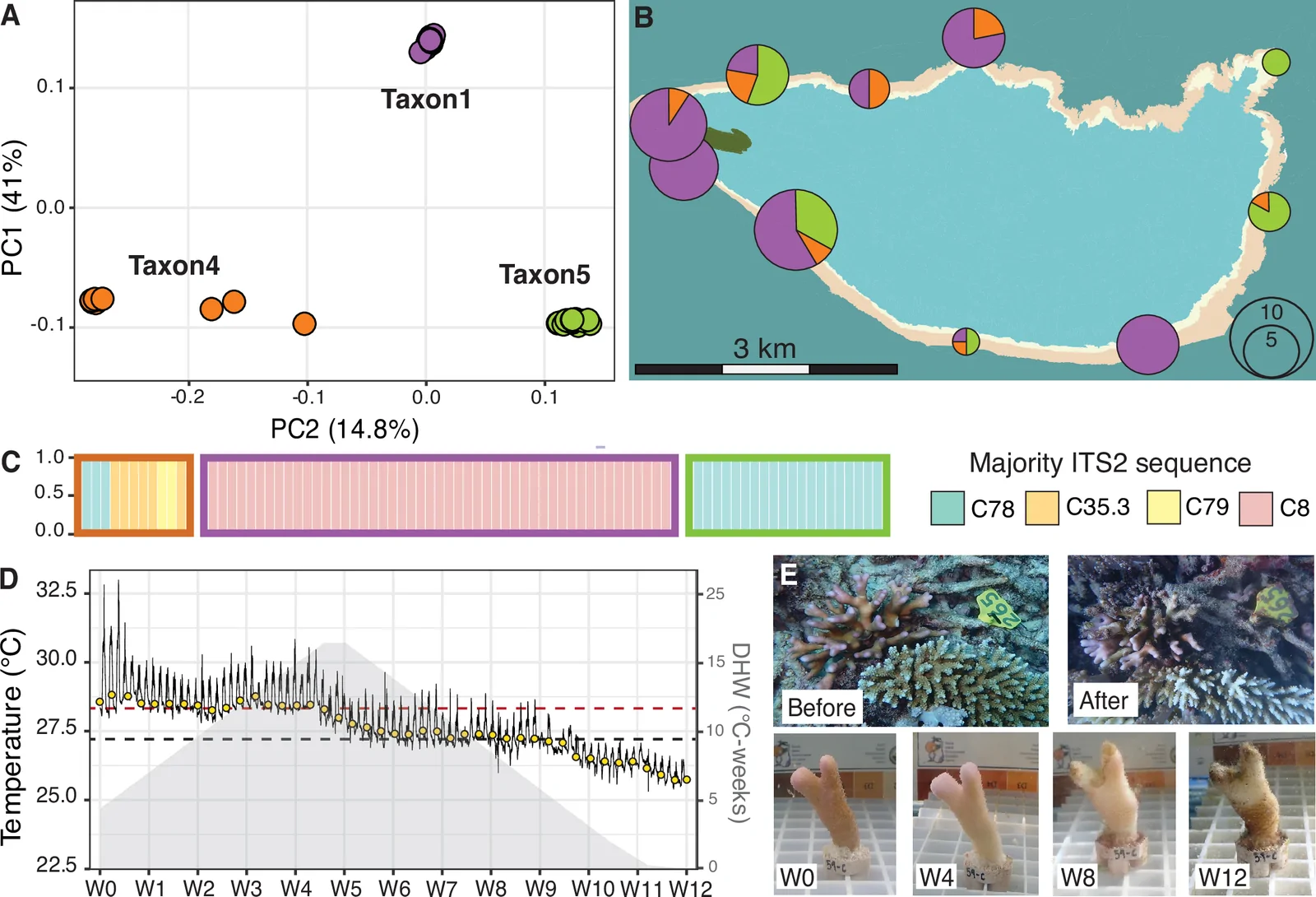
Coral holobiont identities of *S. pistillata* corals at Heron Island Reef and common garden conditions during a heatwave. (A) Principal Component Analysis reveals three taxa, previously identified as *S. pistillata* Taxon1, Taxon4 and Taxon5; PC1 and PC2 axes reversed for visualization. (B) Map showing taxon distribution and sampling effort. (C) ITS2 relative abundance per coral colony, coloured by majority ITS2 sequence. Taxon4 samples ordered to match host differentiation along PC2. (D) Temperature profile in the common garden (average aquarium loggers, black solid line) and on the reef (NOAA CRW 5 km, yellow dots). We show the climatic maximum monthly mean (MMM) (black dashed line), the bleaching threshold (1°C above MMM, red dashed line) and the accumulation of thermal stress over time (grey area). (E) Photographs of a *S. pistillata* coral colony *in situ* before and after the heatwave (upper left coral colony on the image), and a fragment of the same colony in the common garden between week 0 (W0) and week 12 (W12).

### (b) Coral host taxa delineation and *Symbiodiniaceae* community composition assessment

For the 80 coral colonies, whole-genome libraries were prepared and sequenced on a single shared NovaSeq 6000 S4 flowcell (see electronic supplementary material). We used Trimmomatic [51] to quality-filter reads, BWA-MEM [52] to map reads to the *S. pistillata* reference genome (GCA_032172095.1), Samtools v.1.10 [53] to index BAM files and picard [54] to remove PCR duplicates. We used GATK call variants [55] and VCFtools v.0.1.16 [56] to filter the dataset and PLINK v. 2.0 [57,58] to perform identity-by-state and PCA analyses (see electronic supplementary material).

We assessed the symbiotic dinoflagellate community for the 80 coral colonies and for each fragment that survived the thermal stress (*n* = 76) using Internal Transcribed Spacer 2 (ITS2) amplicon sequencing. We used *Symbiodiniaceae*-specific primers [59] and a PCR duplicate for each sample (total of 312 samples), which were sequenced and analysed separately to evaluate variation between PCR reactions (see electronic supplementary material). We used SymPortal [59] to assign ITS2 type profiles based on co-occurring sequence variants. Six samples did not yield any sequence data and were therefore not submitted to SymPortal. For the remaining 606 samples, eight samples had low read counts (<7000 sequence variant reads and/or <6000 ITS2 profile reads; electronic supplementary material) and were discarded from downstream analyses. All duplicates had the same ITS2 profile (Bray–Curtis similarity of profile relative abundance 0.98-1.00), confirming no PCR artefacts (electronic supplementary material, figure S3), and we kept one sample per duplicate pair. Differences in ITS2 sequence variant composition were analysed using pairwise Bray-Curtis dissimilarities and visualized with non-metric multidimensional scaling (NMDS) in the ‘vegan’ R package [60] (see electronic supplementary material). We tested the effect of the host taxonomic identity, sampling location, sampling depth and experimental exposure (i.e. wild colony versus survivor experimental fragment) on ITS2 composition using PERMANOVA (999 permutations; see electronic supplementary material).

### (c) Historical temperature characterization at sampling sites

While colony-level environmental data would have been ideal, obtaining such information was not feasible. Instead, we used modelled data from the GBR1 hydrodynamic model database [61], extracting values based on sampling site coordinates and colony-level depth. We used monthly products and the longest date range available (1 December 2014–1 January 2024), to capture long-term conditions, and the following depth bins: 0.5–2.5, 2.5–5.25, 5.25–9, 9–13 jm. From the temperature data, we calculated four ecologically meaningful variables: mean annual temperature (captures baseline thermal conditions), minimum monthly mean temperature (captures winter cold), maximum monthly mean temperature (captures summer heat) and annual temperature range (captures seasonal variability). Although variation among sites was small (electronic supplementary material, table S1), these differences may be biologically meaningful. These variables were highly correlated (Pearson’s *r* > 0.7), so we performed a PCA and retained the first two axes (temp-PC1 and temp-PC2), which captured 91% and 8% of the variation in the thermal environment (electronic supplementary material, figure S5). Temp-PC1 reflected a longitudinal (east–west) gradient in thermal variability, with higher values indicating warmer temperature maximum, colder temperature minimum and greater temperature range. Temp-PC2 reflected a latitudinal (windward–leeward) gradient in mean temperature, with higher values reflecting lower mean temperature.

### (d) Fragment phenotypic measurements

Every seven days, fragment survival, bleaching status, bleaching percentage and health score were assessed. Survival was classified as alive (entire fragment covered in live tissue), partially dead (part of the fragment without live tissue) or dead (no live tissue). Bleaching status was categorized as no bleaching (no tissue discolouration), partial bleaching (part of the fragment with tissue discolouration) or full bleaching (fragment fully discoloured). Bleaching area was visually estimated and standardized by fragment total surface area, ranging from 0% (no bleaching) to 100% (full bleaching) in 5% increments. Health score was determined using the CoralWatch Health Chart, ranging from 1 (very pale) to 6 (very dark) [62]. Visual scores represent qualitative categories enabling consistent comparisons across fragments and timepoints, rather than exact values.

To capture important aspects of phenotypic responses over time, we calculated four response variables: (i) survival time (i.e. number of weeks a fragment stayed alive, proxy for persistence); (ii) time without bleaching (i.e. number of weeks until the first visual signs of bleaching, proxy for bleaching resistance); (iii) maximum bleaching area per fragment (proxy for bleaching severity); and (iv) recovery time (i.e. number of weeks between lowest health score and the first subsequent increase, proxy for resilience). For (i), (ii) and (iv), a census value of 12 weeks was assigned to fragments that (i) survived until the end of the experiment, (ii) never showed signs of bleaching, and (iii) did not improve health score. Recovery time was calculated only for fragments alive at the end of the experiment that showed signs of paling.

### (e) Survival and bleaching response differences among taxa

All statistical analyses were conducted in R v.3.5.0 [63]. First, differences in survival probability over time among taxa were assessed using a mixed-effects Cox model in the ‘coxme’ R package [64]. We also ran this analysis to investigate survival and bleaching probability over DHW exposure (see electronic supplementary material). We visualized survival trajectories with Kaplan–Meier curves using the ‘survival’ R package [65]. Second, we fitted generalized linear mixed models (GLMMs) using the ‘glmmTMB’ R package [66] for the four traits described above: survival time, time without bleaching, maximum bleaching area and recovery time. In both survival models and GLMMs, we used ‘Taxon’ as a fixed effect, ‘Tank’ (nested within ‘Sump’) and ‘Colony’ (nested within ‘Taxon’) as random effects to account for uncontrolled environmental effects among tanks and colony-level effects, respectively. Any remaining, unexplained variation among replicate fragments of the same colony are estimated as the model residual (see electronic supplementary material). For the GLMMs, significance of fixed effects was assessed using likelihood ratio tests implemented in the ‘car’ R package [67] and estimated marginal means with Tukey *post hoc* pairwise comparisons were obtained using the ‘emmeans’ R package [68].

### (f) Survival and bleaching response differences within taxon among environments

To test whether conspecifics originating from different thermal environments (electronic supplementary material, figure S5) showed different responses, we focused on variation among Taxon1 colonies, since it was the most sampled taxon (see §3). We fitted GLMMs for the four phenotypic traits described above, but to capture variation explained by the historical thermal environment, we used temp-PC1 and temp-PC2 as the fixed effects (see electronic supplementary material). We excluded three sampling sites (BA1, FR1 and Libby’s Lair) with very low sample size (<5 Taxon1 colonies), to limit the potential for unrepresentative samples to bias interpretations. We also omitted two locations (H5A and H5B) where Taxon1 was not sampled. Random effects were again fit to account for data structure as detailed above.

### (g) Survival differences under different heat stress exposures on the reef

If corals on the reef experienced different thermal exposures during the heatwave, survival under *ex situ* common garden conditions should poorly predict *in situ* survival. We assessed this using Cohen’s *κ* (treating common garden versus *in situ* as independent survival raters) and tested the null hypothesis of no agreement (*κ* = 0) using the ‘irr’ R package [55]. Analyses were conducted for all colonies together, and for each taxon separately. Only colonies that could be re-assessed post-heatwave were included. For the common garden data, colony-level survival was defined from the three replicate fragments: ‘alive’ if two or three fragments survived and ‘dead’ if two or three fragments died. The common garden experiment aimed to expose all fragments to a standardized, yet realistic, thermal stress regime; but it is unlikely to exactly replicate the on-reef conditions. This limits conclusions about differences in mean survival *ex situ* versus *in situ* but not about whether survival reflects intrinsic colony responses (i.e. whether colonies with higher *ex situ* survival also had higher *in situ* survival).

## 3. Results

### (a) Genomic data processing and filtering and host taxa delineation

Whole-genome sequencing of the 80 *S*. *pistillata* corals yielded a mean read coverage of 10.2×, 2 021 787 high-quality SNPs. No clones were detected. PCA on 712 815 SNPs linkage-pruned SNPs showed three distinct clusters (figure 1A). Meziere *et al*. [46] discovered four divergent taxa within *S. pistillata* across the GBR (labelled Taxon1, Taxon3, Taxon4 and Taxon5). In the present study, we re-sequenced four representative individuals of Meziere *et al*. [46] for each of the four taxa. Including these representative individuals in our dataset, we assigned the colonies sampled in this study to *S. pistillata* Taxon1 (*n* = 40), Taxon4 (*n* = 11) and Taxon5 (*n* = 20) (electronic supplementary material, figure S6A). Three samples within our dataset appeared taxonomically intermediate (figure 1A), which was not explained by missing data (electronic supplementary material, figure S6B). Downsampling to five samples per taxon resulted in more cohesive grouping of these samples with Taxon4 representative samples (electronic supplementary material, figure S6C), suggesting an artefact of uneven sample sizes among taxa. However, ADMIXTURE analysis showed that one of these individuals might be of hybrid origin (electronic supplementary material, figure S7). We assigned this sample to Taxon4 based on its PCA position and higher Taxon4 ancestry, but we also confirmed that excluding this sample did not affect downstream statistical results. Notably, we sampled at least two taxa at most (7/10) sites (figure 1B) with no clear partitioning of taxa across water depth (electronic supplementary material, figure S8).

### (b) Symbiodiniaceae community composition assessment

We identified four ITS2 type profiles across the 80 original colonies, all within *Symbiodiniaceae* Clade C: C8/C8a-C1-C42.2-C42e-C8e-C42ae (*n* = 49 Taxon1 coral hosts), C78/C78c-C1-C3lk-C3gk-C1re-C78d (*n* = 2 Taxon4 and *n* = 21 Taxon5 coral hosts), C35.3/C35.1/C35a-C54l (*n* = 6 Taxon4 coral hosts) and C79/C54l-C79b-C79a-C40ap-C54m (*n* = 2 Taxon4 coral hosts) (figure 1C, electronic supplementary material, figures S3 and S4). These are hereafter referred to by their majority ITS2 sequence (i.e. C8, C78, C35.3 and C79). Each coral was dominated by an ITS2 type profile (i.e. no mixed communities), and all except C78 were highly host-taxon specific. Intriguingly, the only three surviving Taxon4 fragments were from colony #66, which hosted C78 symbionts and was also the only Taxon4 colony found alive on the reef post-heatwave. ITS2 sequencing of the 76 surviving experimental fragments revealed stable symbiont communities, with only four fragments that exhibited different ITS2 profiles pre- and post-heat stress: the three fragments of Taxon1 colony #49 switched from C8 to C78, and one fragment of Taxon1 colony #59 switched from C8 to a mixed community of C8 and A1er (electronic supplementary material, figure S3). These fragments never showed signs of bleaching.

NMDS of Bray–Curtis dissimilarities revealed clear structuring of ITS2 sequence variants by SymPortal type profiles (electronic supplementary material, figure S4), with some additional differentiation by sampling depth within clusters. PERMANOVA confirmed that ITS2 composition was significantly structured by the host taxonomic identity (*R*² = 0.184, *p* = 0.006), while sampling location (*R*² = 0.063, *p* = 0.307), depth (*R*² = 0.005, *p* = 0.099) and experimental exposure (*R*² = 0.005, *p* = 0.008) had comparatively weak contributions.

### (c) Survival and bleaching response differences among host taxa

One hundred and sixty-four out of 240 fragments (68%) were dead at the end of the experiment and 39 (16%) had partial mortality. Survival differed among taxa, with all three fragments dying in 25/49 (51%) Taxon1 colonies, in 10/11 (90%) Taxon4 colonies, and in 6/20 (30%) Taxon5 colonies (figure 2A). Survival analyses supported taxon-specific responses with statistical significance (*χ*² = 15.1, *p* = 0.0005, electronic supplementary material, table S3), with lowest mortality risk in Taxon5, and highest mortality risk for Taxon4 (figure 2B). Survival differed significantly between Taxon4 and the other taxa before peak DHW (*χ*² = 36.3, *p* = 1.3e-08; electronic supplementary material, figure S9A) and among all taxa after peak DHW (*χ*² = 14.7, *p* = 0.0006; electronic supplementary material, figure S9B), reflecting the continuing mortality of Taxon1 despite cooling water temperatures. These differences are supported by significant differences among taxa in the average number of weeks they remained alive in the common garden (*χ*² = 41.6, *p* < 0.001; figure 2C, electronic supplementary material, tables S3 and S4).

**Figure 2.**
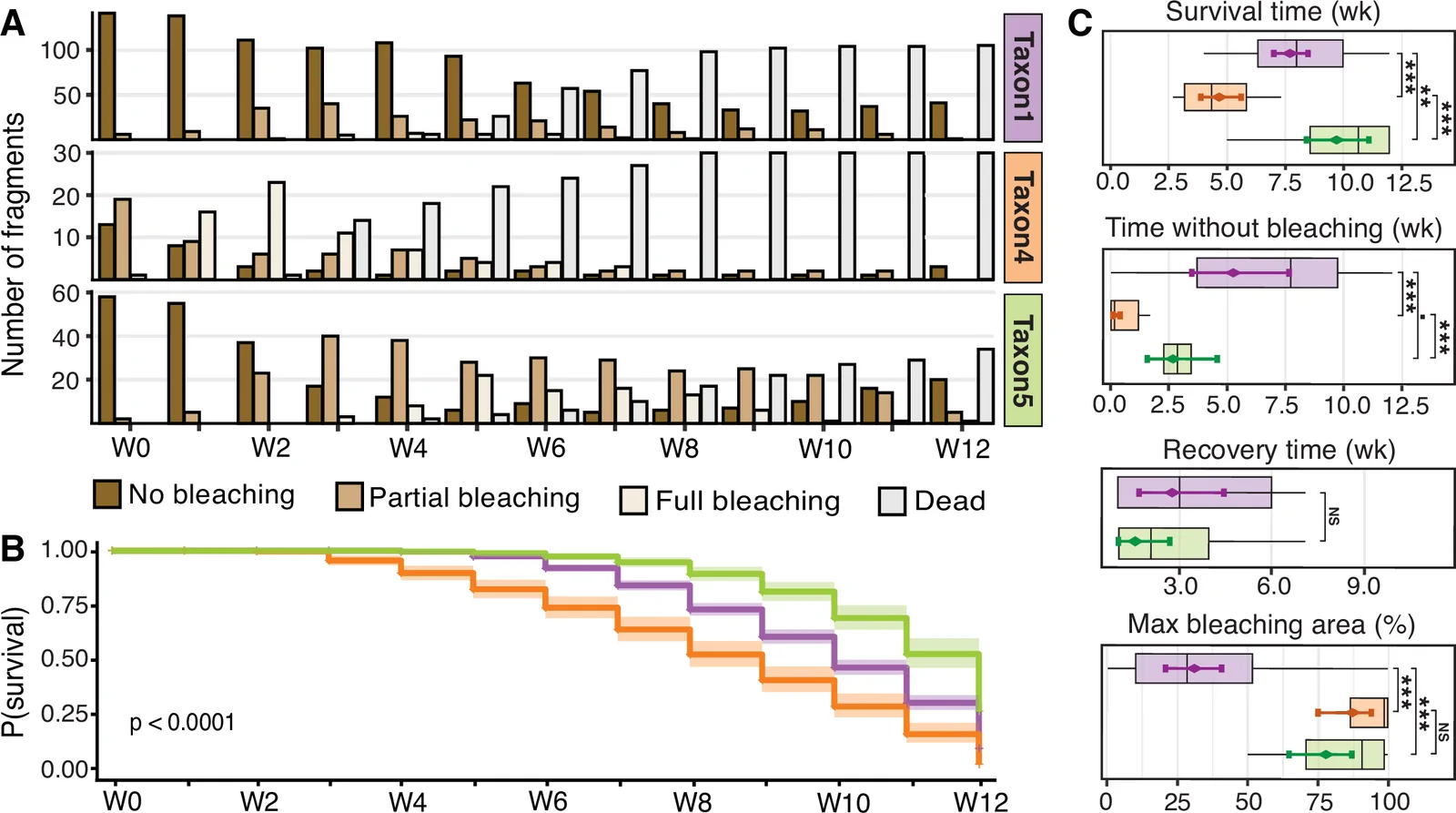
Survival and bleaching responses of *S. pistillata* taxa during 12 weeks (W0–W12) of thermal stress in the common garden experiment. For each taxon, we show (A) the number of alive fragments by their bleaching status (full, partial, none) and dead fragments, (B) Kaplan–Meier curves of colony survival probability and (C) the distributions of colony-level averages (across the three fragment replicates) for the number of weeks a colony was alive, the number of weeks a colony did not show signs of bleaching, the number of weeks it took a colony’s health score to increase after reaching its lowest value, and a colony’s maximum bleached area. Recovery time for Taxon4 is not shown because only fragments from only one Taxon4 colony survived. Diamonds represent model-estimated marginal means (with 95% confidence intervals) and significance of pairwise differences is indicated as: ‘NS’ for *p*  >  0.1, ‘*.’* for *p*  <  0.1, ‘*’ for *p*  <  0.05, ‘**’ for *p*  <  0.01, ‘***’ for *p*  <  0.001.

We observed bleaching (full or partial) in 177 (74%) fragments over the course of the experiment and bleaching recovery occurred in 46 (19%) fragments (figure 2A). Bleaching varied significantly by taxon (*χ*² = 67.8, *p* < 0.001), as did the maximum bleaching area (*χ*² = 63.6, *p* < 0.001) (figure 2C). Notably, while Taxon5 had the highest survival, it bleached faster and more extensively than Taxon1 (electronic supplementary material, tables S3 and S4, figure S9C,D). Finally, while the recovery time was slightly faster in Taxon5 fragments, we found no significant difference in recovery time between Taxon1 and Taxon5 (figure 2C, electronic supplementary material, table S3). We could not estimate recovery times in Taxon4 because only three fragments (from the same colony) survived until the end of the experiment. Statistical significance in survival and mixed model analyses was unaffected by the exclusion of the putative hybrid sample (see electronic supplementary material).

### (d) Survival and bleaching response differences within taxon among environments

While temp-PC1 and temp-PC2 had no significant effect on survival time among Taxon1 colonies (temp-PC1: *χ*² = 1.35, *p* = 0.24; temp-PC2: *χ*² = 0.83, *p* = 0.36), there was weak evidence that their bleaching responses may vary along temp-PC1, which captures thermal variability and more extreme temperatures (time without bleaching: *χ*² = 3.21, *p* = 0.07; maximum bleaching area: *χ*² = 2.78, *p* = 0.09). Specifically, faster bleaching and more extensive bleaching were marginally associated with higher temperature variability (i.e. higher temp-PC1 scores). There was no evidence that bleaching responses varied along temp-PC2, which captures mean temperature (time without bleaching: *χ*² = 0.31, *p* = 0.57; maximum bleaching area: temp-PC2: *χ*² = 0.74, *p* = 0.38).

### (e) Survival differences under different heat stress exposures on the reef

Following the heatwave (three months after initial sampling), we located 53 (66%) of the 80 tagged source colonies. Of these 53, 12 (23%) were alive, 13 (24%) had partial mortality and 28 (53%) were dead (figure 3B, electronic supplementary material, figure S10). Of the 25 living colonies, 23 appeared healthy (no bleaching) and two showed partial bleaching. On-reef colony survival differed markedly among taxa (figure 3A): nearly all Taxon4 re-located colonies (6/7) were dead, whereas 48% (15/31) of Taxon1 and 60% (9/15) of Taxon5 re-located colonies were alive, consistent with survival patterns under common garden conditions.

**Figure 3.**
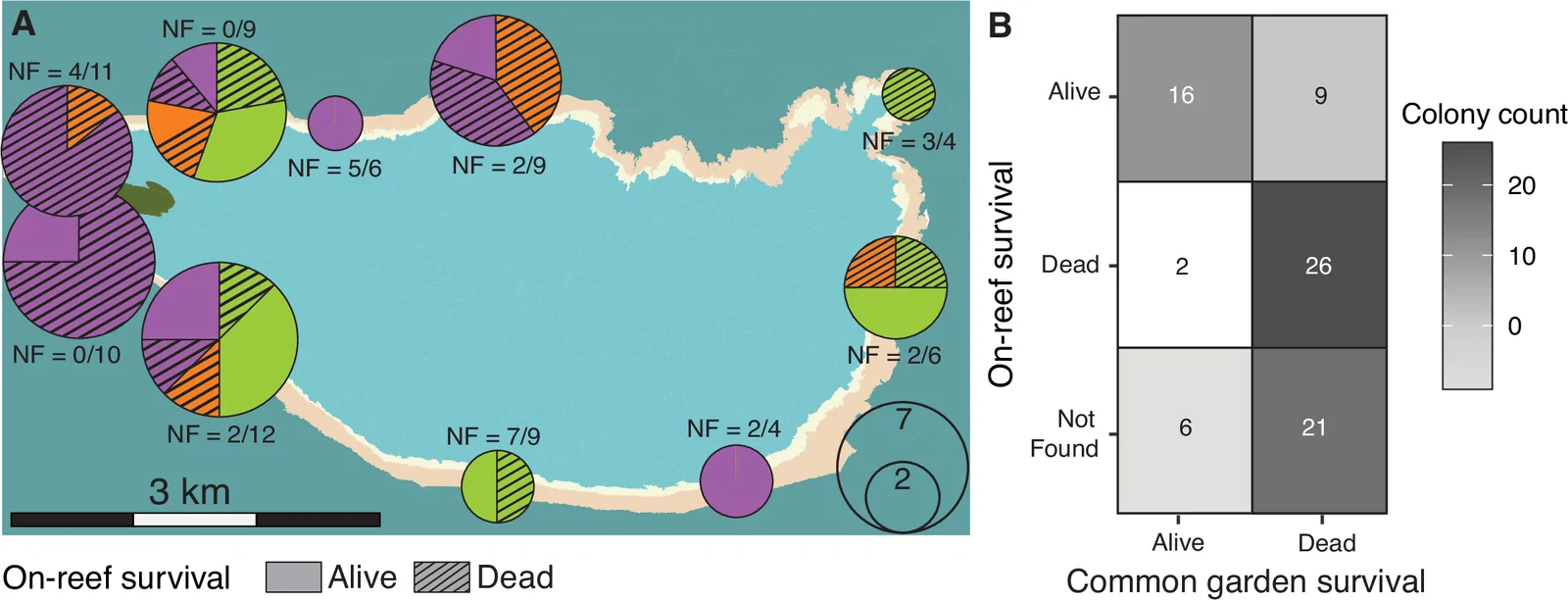
On-reef survival of tagged *S. pistillata* colonies following the heatwave. (A) Map showing survival of the sampled colonies for each taxon (purple, orange and green for Taxon1, Taxon4 and Taxon5, respectively). Dead colonies are represented with stripes and alive colonies with block colours; NF indicates the number of colonies that we could not find post-heatwave. (B) heatmap showing the relationship between survival of the 80 colonies on-reef following the heatwave (rows) and in the common garden experiment (columns). Each tile shows the number of colonies for each combination (e.g. of the 25 colonies that were alive *in situ* post-heatwave, 16 were categorized as alive in the common garden and 9 as dead).

We found a significant association between colony survival under common garden conditions and on-reef survival (*κ* = 0.171, *z* = 4.74, *p* = 0.00018) with a Spearman’s correlation coefficient of *ρ* = 0.62 (figure 3B). Interestingly, most colonies that could not be re-located on the reef had dead fragments in the common garden. Dead colonies in the field, likely overgrown with macroalgae, may have been difficult to detect, explaining our inability to find them. The association between common garden and on-reef survival was also significant within each taxon (Taxon1: *κ* = 0.121, *z* = 1.97, *p* = 0.049; Taxon4: *κ* = 0.462, *z* = 2.65, *p* = 0.008; Taxon5: *κ* = 0.130, *z* = 2.37, *p* = 0.018; electronic supplementary material, figure S10). This strong association of survival outcomes suggests that differential exposure to heat stress across the reef likely contributes little to differential survival.

## 4. Discussion

In this study, we explored sources of thermal tolerance variation among coral colonies of the *S. pistillata* species complex during a marine heatwave at Heron Island Reef, Australia. Combining a common garden experiment and *in situ* monitoring, we found high concordance between on-reef survival and common garden survival, suggesting a negligible effect of thermal stress exposure differences among reef habitats. This finding also confirms that the common garden observations likely provided biologically meaningful insights into on-reef responses. Instead, we found most bleaching and survival differences among coral colonies reflected the intrinsic sensitivity of closely related taxa and their associated symbionts. Unexpectedly, we found some evidence for higher within-species thermal sensitivity in corals inhabiting historically warm and variable habitats.

### (a) Cryptic taxa differ in their bleaching responses and survival

Our study was the first to pair a coral common garden experiment with *in situ* reef observations during a marine heatwave, thereby testing intrinsic differences in thermal stress response. Under common garden conditions, the three *S. pistillata* taxa showed different bleaching and survival patterns (figure 2), demonstrating that observed inter-specific differences reflect sensitivity differences among these taxa, not differential heat stress exposure in their native habitat. Importantly, we also show that survival on the reef mirrored experimental survival (figure 3). Across taxa, studies have revealed that previously presumed generalist species are in fact complexes of specialist cryptic species (reviewed in [17]). Our findings add to this growing body of literature and lend support to previously described response diversity among cryptic coral species (e.g. [18,19,69]). Variation in how cryptic species respond to environmental pressure can have far-reaching ecological consequences [70]. Under recurring climate extremes, it is plausible that more susceptible taxa (such as *S. pistillata* Taxon4) could be gradually replaced by more resilient ones (such as *S. pistillata* Taxon5). Conversely, if all cryptic species within a species complex showed similar environmental susceptibility, entire morphological groups could be at risk of extinction. Importantly, if cryptic species are not recognised, assessments of ecological resilience may be biassed, and substantial losses of species and genetic diversity could go undetected.

While cryptic species are often reported to occupy different environmental niches, we found no strong patterns of spatial separation among the three *S. pistillata* taxa sampled in our study (figure 2B). We do note, however, that *S. pistillata* Taxon5 was found at the fewest sites, and at most of these sites (4 out of 5) it was found at shallower depths (electronic supplementary material, figure S8). Thus, our relatively small sample numbers might dampen our power to detect habitat preferences, and more extensive sampling (including at additional reefs), with a focus on sampling across the depth gradient, could reveal habitat specialization.

Notably, in addition to differences in ultimate survival outcome among taxa, we also identified differences in the trajectories of bleaching and survival. While Taxon1 and Taxon5 colonies had similar survival trajectories during the early heat stress accumulation phase (i.e. before peak DHW, electronic supplementary material, figure S9), they showed different long-term outcomes, with Taxon1 exhibiting higher mortality than Taxon5 after peak DHW (electronic supplementary material, figure S9). These results imply that short-term (i.e. a few days or weeks) observations, either *in situ* or under experimental exposure, may not be reliable predictors of long-term resilience. This finding is aligned with observations of delayed impacts of heat stress in other coral species, such as increased mortality in heated *A. hyacinthus* coral fragments up to 6 month post-heat stress [71]. Thus, although acute heat stress assays have become popular to test coral thermal limits (e.g. [72,73]), observations under more realistic conditions show that responses during a recovery phase might shape the final outcome of an extreme event.

### (b) Strong host–symbiont specificity limits separation of host and symbiont effects

Coral photosynthetic dinoflagellates vary extensively in how they are transmitted among hosts, which can influence how host-specific they may be. Here we found strong host-symbiont specificity (figure 2C), consistent with expectations for a brooding coral species with maternal symbiont transmission [74,75]. Because host and symbiont taxon identity were highly correlated, we could not estimate their relative contributions to the observed thermal stress responses. However, our results appear consistent with an earlier study of *S. pistillata* corals at Heron Island Reef, which reported that colonies hosting C8 (here, exclusive to Taxon1) and C78 (here, found in all Taxon5) *Symbiodiniaceae* species bleached less than those associated with C79 and C35 (here, exclusive to Taxon4) [48].

Interestingly, the only surviving Taxon4 colony (both *ex situ* and on the reef) harboured C78 symbionts and one surviving Taxon1 colony (both *ex situ* and on the reef) switched from C8 to C78 during the common garden. C78 symbionts were associated with all colonies of Taxon5, which showed the highest survival rate, suggesting that this symbiont may provide greater thermal tolerance. Additionally, the most sensitive Taxon4 had the greatest *Symbiodiniaceae* diversity, which may reflect the re-acquisition of different symbiont taxa during past bleaching events. This shuffling could have occurred during the lifespan of the sampled colonies or in a previous generation if shuffled symbionts were inherited via vertical symbiont transmission [76]. Overall, these observations suggest that *Symbiodiniaceae* community composition may be a key contributor to response variation among *S. pistillata* holobionts, consistent with the broader role of microbiomes in host fitness (e.g. [77–79]). Manipulative experiments associating each host taxon with different symbionts prior to heat stress could help clarify the relative contributions of the host and the symbionts.

### (c) Conspecifics from historically warmer habitats show greater thermal susceptibility

Significant correlation between common garden and *in situ* survival demonstrated that intrinsic differences among coral colonies, not differential heat exposure on the reef, contributed to thermal stress response variation. However, intrinsic factors may themselves reflect historical heterogeneity in environmental experiences. Across a wide range of taxa, there is extensive evidence that organisms can be locally adapted or long-term acclimatized to micro-habitats (reviewed in [7]. This has also been evidenced in some coral species, where conspecifics sampled at distinct reef habitats or depths differed in their tolerance to heat stress (e.g.[37,80]). Here, we investigated whether historical thermal conditions at our sampling site could explain phenotypic variation among Taxon1 colonies under common garden conditions. While weak, we detected evidence for faster and more-severe bleaching for Taxon1 coral colonies originating from sites that experience greater seasonal variability and/or the warmest monthly peaks (Western windward side of the reef). These patterns also aligned with greater Taxon1 colony mortality *in situ* at Heron Bommie and Coral Garden (figure 3A; electronic supplementary material, figure S5). However, these observations are inconsistent with local adaptation or adaptive long-term acclimatization, where corals originating from warmer or more thermally variable habitats should show higher survival and reduced bleaching.

These counterintuitive results may arise because adaptation to historical temperature averages may not be beneficial under present-day and future acute thermal stress events. Additionally, the strong host–symbiont fidelity in this species complex limits the potential for acquiring more heat-tolerant symbionts for corals that have previously experienced bleaching, a mechanism proposed to enhance thermal tolerance in other species [22]. A complementary explanation is that chronic exposure to sub-lethal temperatures can induce carryover effects in the form of passive damage accumulation [32,81]. Thus, *S. pistillata* corals from warmer habitats might be living at their upper thermal tolerance limits and have reduced capacity to cope with additional heat stress during a heatwave. Such physiological carryover effects have been documented in a range of organisms [82,83] and previous observations of more severe bleaching in historically warmer coral reef habitats [26] are consistent with our observations. Future investigations of coral survival during heatwaves should therefore not assume that corals are locally adapted and should consider prior physiological stress. This would extend our understanding of this phenomenon and determine how it may impact our predictions of future biodiversity changes.

## 5. Conclusion

We found that intrinsic sensitivity, but not differential exposure, was key in shaping *S. pistillata* coral thermal tolerance. Cryptic species identity and their associated symbionts strongly influenced colony-level bleaching and survival, responses under common garden conditions predicted survival on the reef and habitat thermal history may contribute further variation among individuals. Our findings highlight the importance of separating intrinsic from extrinsic factors to understand why individuals are differentially impacted during extreme climatic events. Such insights are critical to improve predictions of future losses of species and genetic diversity.

## Supplementary Material



## Data Availability

Raw sequence data are available on the NCBI Sequence Read Archive under the BioProjects PRJNA1358725 (host whole-genome sequencing) and PRJNA1441812 (Symbiodiniaceae ITS2 sequencing). Metadata for sampled coral colonies is available on GEOME under the GUID https://n2t.net/ark:/21547/Giu2. Scripts and data to reproduce analyses and figures are available on Zenodo [84]. Supplementary material is available online [85].

## Supplement

Electronic supplementary material is available online at https://doi.org/10.6084/m9.figshare.c.8472232.
